# Reappraisal of a historical porfimer sodium photodynamic therapy study for vascular restenosis: Efficacy, high procedural mortality, and methodological insights from a rabbit balloon-injury model

**DOI:** 10.1371/journal.pone.0350675

**Published:** 2026-06-22

**Authors:** Ai-Hsien Adams Li, Yuan Hung Liu, Ya-Hui Tsai, Chi-Yu Yang, Kuan-Ming Chiu

**Affiliations:** 1 Cardiovascular Center, Far Eastern Memorial Hospital, New Taipei, Taiwan; 2 College of Informatics, Yuan Ze University, Taoyuan City, Taiwan; 3 College of Medicine, National Taiwan University, Taipei, Taiwan; 4 School of Nursing, Yuan-Ze University, Taoyuan City, Taiwan; 5 Laboratory Animal Center, Far Eastern Memorial Hospital, New Taipei, Taiwan; IPSurface Canada Inc, CANADA

## Abstract

**Background:**

Restenosis driven by intimal hyperplasia remains a major limitation of peripheral arterial intervention. Photodynamic therapy (PDT) using porfimer sodium (Photofrin®) demonstrated promising preclinical efficacy in the 1990s; however, its translation into clinical vascular practice has been stalled. We reappraised a historical pilot dataset to evaluate both biological efficacy and procedural feasibility.

**Methods:**

In a rabbit model of bilateral femoral artery balloon injury (10 animals initiated), one artery per animal received intraluminal PDT (630 nm, ~ 5.6 J/cm²) 48 h after intramuscular porfimer sodium administration (2 mg/kg), while the contralateral artery served as an internal control. Histomorphometric analysis of the intima-media ratio (IMR) was performed 21 days after injury.

**Results:**

The procedural feasibility of this approach is severely limited. Only three of the ten animals yielded analyzable paired arterial samples because of high intraoperative mortality (40%) and postoperative complications. In surviving animals, PDT-treated arteries demonstrated a consistent and marked suppression of neointimal hyperplasia compared with controls (IMR 0.29 ± 0.07 vs. 1.65 ± 0.55; *P < 0.001*), corresponding to a median IMR reduction of 82%.

**Conclusions:**

This reappraisal confirms the potent biological effects of porfimer sodium-mediated PDT on neointimal hyperplasia. However, the principal contribution of this study lies in the transparent documentation of critical translational barriers, particularly the unsustainable procedural mortality associated with this model of bilateral injury. By providing detailed dosimetry parameters and a candid feasibility analysis, this study offers methodological insights to guide the design of safer and more translatable preclinical vascular PDT studies in the future.

## 1. Introduction

Percutaneous transluminal angioplasty (PTA) with or without stenting is the cornerstone of treatment for symptomatic peripheral arterial disease (PAD), particularly in the femoropopliteal segment [1,2]. However, the long-term efficacy of endovascular interventions for PAD remains compromised by restenosis, which occurs in 30–40% of cases within the first year, a rate significantly higher than that observed in coronary interventions [3,4]. This disparity persists despite considerable advances in device technology and the development of pharmacological adjuncts for its treatment.

Restenosis is primarily driven by intimal hyperplasia (IH), a pathological process characterized by the migration and proliferation of vascular smooth muscle cells (VSMCs) triggered by endothelial injury during intervention [5,6]. Although drug-eluting stents have demonstrated efficacy in the coronary arteries, their performance in peripheral vessels has been inconsistent, with some studies showing minimal benefit over bare-metal stents [7,8]. The unique biomechanical environment of peripheral arteries, including greater vessel length, more complex anatomical geometry, and exposure to repetitive flexion and compression, contributes to these differential outcomes [9].

The persistent challenge of peripheral arterial restenosis has motivated investigations into alternative approaches targeting the fundamental biology of VSMC proliferation. Photodynamic therapy (PDT) is a mechanistically distinct strategy that generates localized cytotoxic effects via light activation of photosensitizing agents. Upon exposure to specific wavelengths, photosensitizers produce reactive oxygen species (ROS) that selectively induce apoptosis and necrosis in metabolically active, proliferating cells while sparing quiescent tissues when dosimetry is appropriately controlled [10,11].

Several preclinical studies have explored PDT for vascular applications, demonstrating proof-of-concept suppression of neointimal formation with photosensitizers, including indocyanine green and 5-aminolevulinic acid derivatives [12–14]. Porfimer sodium (Photofrin®), the first FDA-approved photosensitizer for oncological applications, possesses favorable pharmacokinetic properties, including preferential accumulation in proliferating tissues and well-characterized safety profiles from extensive clinical use. However, systematic investigations of porphyrin-mediated vascular PDT remain limited.

The rabbit femoral artery balloon injury model has served as a mainstay in vascular research because of its anatomical similarity to human peripheral vessels and its reproducible induction of neointimal hyperplasia [15]. However, this model has inherent technical challenges. Bilateral femoral artery access can induce acute limb ischemia and systemic inflammatory responses, potentially affecting both animal welfare and experimental outcomes [16,17]. These considerations, though often underreported, are critical for interpreting the validity of the study and informing future protocol modifications.

### 1.1. Study objectives

The primary objective of this pilot feasibility study was to evaluate the photobiological efficacy of porfimer sodium-mediated PDT in suppressing neointimal hyperplasia following balloon injury using a within-subject paired-control design to minimize biological variability. The secondary objectives were as follows.

1. Establishing clinically relevant photodynamic dosimetry parameters for vascular applications
2. Documenting procedural challenges and mortality rates to inform future protocol optimization
3. Assessing the consistency of treatment effect across individual subjects
4. Providing foundational data to support further investigation of PDT for peripheral arterial restenosis prevention

Given the ongoing clinical challenge of restenosis after peripheral interventions and the limited success of coronary-derived technologies in this anatomical territory, we believe that these findings warrant renewed attention to photodynamic approaches for treating vascular diseases.

Thus, the present manuscript does not claim novelty in concept but provides a detailed retrospective methodological analysis of a pilot study that encountered severe translational roadblocks. Its primary aim is to extract and disseminate lessons regarding feasibility challenges and model optimization, providing a transparent case study for the translational research community.

## 2. Materials and methods

### 2.1. Animal model and photosensitizer

All procedures were approved by the Institutional Animal Care and Use Committee of Far Eastern Memorial Hospital (FEMH 92-D-008). Ten male New Zealand white rabbits (3–4 kg; BioLASCO Taiwan Co., Ltd.) (one rabbit per cage for close monitoring) were used in this study. The photosensitizer, porfimer sodium (Photofrin®; Pinnacle Biologics), was intramuscularly administered at a dose of 2.0 mg/kg 48 h before surgery. The study was conducted in strict accordance with the Guide for the Care and Use of Laboratory Animals (NIH) and the Taiwan Animal Protection Act. As this study involved no human participants, no human consent was required. All surgeries were performed under general anesthesia, and all efforts were made to minimize suffering.

### 2.2. Surgical procedure and balloon injury

General anesthesia was induced using intramuscular xylazine hydrochloride (5 mg/kg) and ketamine hydrochloride (35 mg/kg). Depth was assessed by loss of pedal withdrawal and maintained with a supplemental half-dose if spontaneous movement was observed. Intra-arterial pressure or capnography was not used, which may have contributed to the high mortality rate. These monitoring modalities are now recognized as essential for detecting early cardiovascular compromise during major vascular procedures in rabbits, and their absence represents a correctable limitation. Body temperature was maintained using a homeothermic blanket. Postoperative analgesia was provided with buprenorphine (0.03 mg/kg, IM, every 8–12 hours for 48 hours). Animals were monitored twice daily for signs of distress, wound dehiscence, or hindlimb ischemia. Humane endpoints were defined as signs of severe respiratory distress, sustained limb necrosis, or inability to ambulate and access food and water.

The femoral arteries were surgically exposed bilaterally. A 2.0 mm angioplasty balloon catheter was introduced and inflated to 14 atm for 60 s to induce endothelial injury and then withdrawn from the artery.

### 2.3. Photodynamic Therapy (PDT)

In each animal, an injured artery was randomly selected for PDT. A 425-µm optical fiber with a cylindrical diffuser tip (25 mm) was inserted into the target artery of the rabbits.

**Laser Dosimetry Validation** with fluence rate measurement and optical depth (λ = 630 nm, penetration ≈ 2–3 mm): Light from a 630 nm diode laser (Biolitec Diomed 630 PDT) was delivered at a total power of 2.0 W through a 25-mm cylindrical diffuser, producing an estimated power density of ≈ 80 mW/cm² at the arterial wall. The irradiation lasted 70 s, giving a total fluence of ≈ 5.6 J/cm². (All parameters were verified using a calibrated photodiode power meter before each exposure.)

The contralateral injured artery did not receive light treatment and served as an internal control. All arteries were repaired using 7−0 polypropylene sutures.

The animals were monitored postoperatively and provided supportive care. Euthanasia was performed humanely under deep anesthesia in accordance with institutional guidelines. Specifically, euthanasia at the 21-day endpoint was performed by intravenous pentobarbital overdose (100 mg/kg, via marginal ear vein) under deep anesthesia (xylazine/ketamine as above), consistent with the AVMA Guidelines for the Euthanasia of Animals.

### 2.4. Histological and morphometric analysis

After a 21-day survival period, the rabbits were euthanized, and the femoral arteries were harvested, fixed in formalin, and embedded in paraffin. Cross-sections (5 µm) were stained with hematoxylin and eosin (H&E). Morphometric analysis was performed by an investigator blinded to the treatment groups. The intima-media ratio (IMR) was quantified by measuring the distance from the endothelium to the internal elastic lamina divided by the distance from the internal elastic lamina to the adventitia (ET-IEL/IEL-AD) in three locations per artery.

All animal procedures were performed in accordance with the ARRIVE 2.0 guidelines (S1 File).

### 2.5. Quantification and statistical analysis

Morphometric measurements were performed using ImageJ software (NIH) [18]. Statistical analyses were conducted using R software (R Foundation for Statistical Computing). Owing to the small sample size and non-normal distribution of data, the Wilcoxon signed-rank test was used to compare the IMR values between the PDT and control groups and to report the exact test statistics. The Kruskal-Wallis test was used to assess differences among animals. Statistical significance was set at P < 0.05. In addition, we reported Cohen’s d value for the effect size confidence interval (CI).

Given the paired nature of the data (bilateral arteries within each animal), the primary comparison of IMR between the PDT and control arteries was performed using the paired Wilcoxon signed-rank test. The effect size was calculated as r = Z/√N. The sample size was determined based on pilot study design principles to detect large effect sizes (Cohen’s d > 1.5).

## 3. Results

### 3.1. Animal survival and feasibility

Of the ten rabbits, four died intraoperatively due to suspected anesthesia-related complications, and one died in the third week due to a suspected postoperative infection. Among the five survivors, two were excluded because of distal occlusion of the target vessel. Thus, three animals (Rabbits C, D, and E) provided paired arterial samples (n = 3 PDT, n = 3 control) for the final analysis. This high attrition rate was a critical limitation of our initial feasibility study. Importantly, all four intraoperative deaths occurred prior to PDT delivery, indicating that procedural mortality was attributable to bilateral balloon injury and anesthesia burden rather than to the PDT protocol itself. A structured attrition summary is presented in Table 2. Formal gross post-mortem organ evaluation was not performed on animals that died intraoperatively or early postoperatively — a significant limitation of this historical pilot study that precludes definitive cause-of-death attribution. Future investigators should incorporate mandatory post-mortem protocols.

### 3.2. Efficacy of PDT on neointimal hyperplasia

Histological examination confirmed markedly reduced neointimal thickening in PDT-treated arteries compared to internal controls (Fig 1–2, and Fig 3).

**Fig 1:**
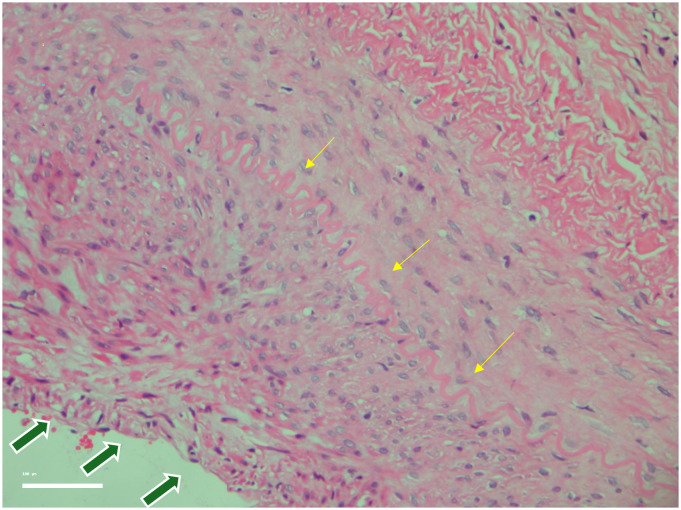
Histological evidence of photodynamic therapy-mediated suppression of neointimal hyperplasia (Animal E) — Control artery. Representative hematoxylin and eosin-stained cross-section of the balloon-injured femoral artery (100 µm scale bar). IEL: internal elastic lamina (yellow arrow). Vascular endothelium is indicated by green arrows. The control artery shows marked intimal thickening between endothelium (green arrows) and internal elastic lamina (yellow arrows), occupying approximately 65% of the vessel wall cross-section.

**Fig 2:**
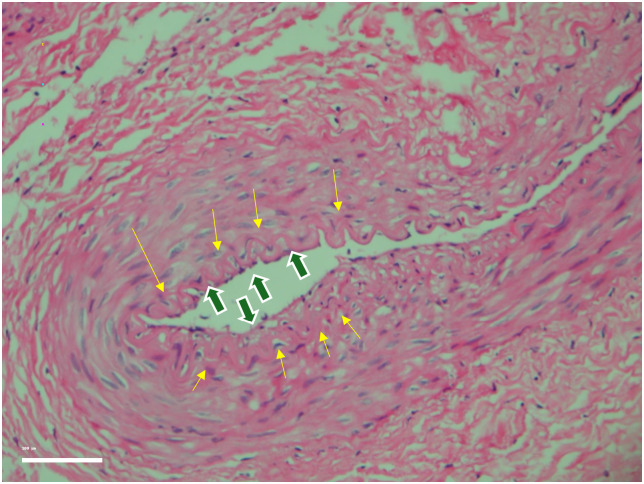
Histological evidence of photodynamic therapy-mediated suppression of neointimal hyperplasia (Animal E) — PDT-treated artery. Representative hematoxylin and eosin-stained cross-section of the balloon-injured femoral artery (100 µm scale bar). IEL: internal elastic lamina (yellow arrow). Vascular endothelium is indicated by green arrows. The PDT-treated artery demonstrates markedly reduced neointimal proliferation and preserved media structure compared with the contralateral control.

**Fig 3:**
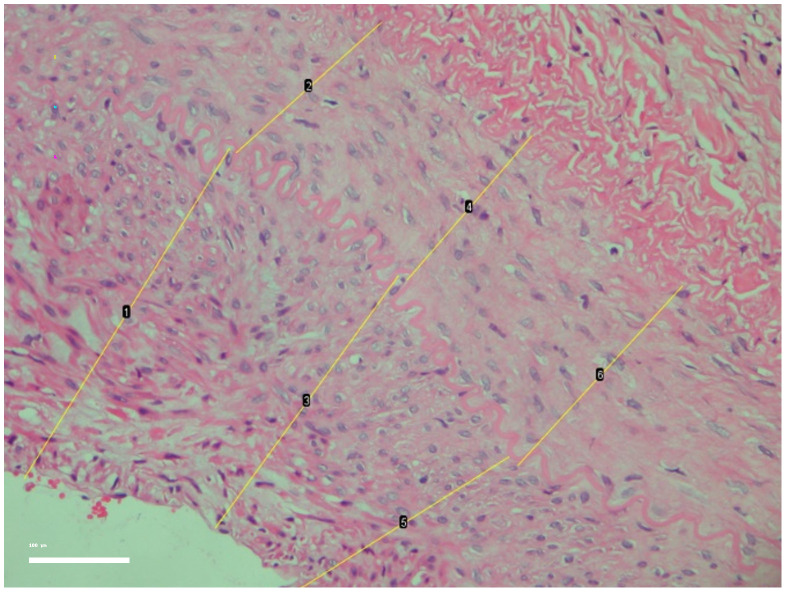
Schematic overlay illustrating morphometric measurement of the Animal E control-side arterial section. Lines 1, 3, and 5 represent intimal thickness (distance between endothelium and internal elastic lamina, IEL); lines 2, 4, and 6 represent media thickness (distance between IEL and adventitia). The intima-media ratio (IMR) is calculated as the mean intimal thickness divided by the mean media thickness across three measurement locations per artery.

Quantitative analysis demonstrated a significantly lower intima-media ratio (IMR) in PDT-treated arteries than in contralateral controls (0.291 ± 0.072 vs. 1.647 ± 0.554; paired Wilcoxon signed-rank test: T = 0, z = −2.88, P = 0.0004; n = 3 pairs).

Visualization information is shown in Fig 4 and Table 1.

**Table 1:** Animal Attrition Summary.

Animal	Outcome	Timing	Included in Analysis
A	Intraoperative death	During surgery (before PDT)	No
B	Intraoperative death	During surgery (before PDT)	No
C	Survived; analyzable paired sample	Survived to Day 21	Yes
D	Survived; analyzable paired sample	Survived to Day 21	Yes
E	Survived; analyzable paired sample	Survived to Day 21	Yes
F	Intraoperative death	During surgery (before PDT)	No
G	Intraoperative death	During surgery (before PDT)	No
H	Postoperative death (Week 3)	Week 3 (suspected infection)	No
I	Excluded: distal vessel occlusion	Technical exclusion	No
J	Excluded: distal vessel occlusion	Technical exclusion	No

**Fig 4:**
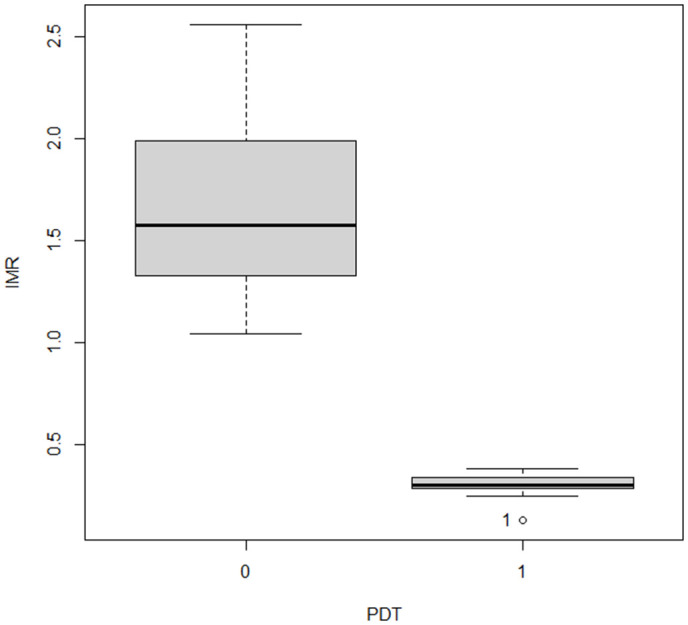
Box and whisker plot of PDT (labeled 1) vs. control (labeled 0). P = 0.0004038 by paired Wilcoxon signed-rank test.

The effect size was large (Cohen’s d = 2.8), with a bias-corrected bootstrap 95% confidence interval of 1.1–4.5.

No significant inter-animal differences were observed (Kruskal-Wallis P = 0.801).

### 3.3. Individual animal responses

The three animals exhibited consistent responses to PDT. Specifically, Animal C showed a reduction in IMR from 2.31 to 0.25, an 89% reduction. Animal D showed a reduction in IMR from 1.19 to 0.40, a 66% decrease. Animal E experienced a reduction in IMR from 1.44 to 0.23, an 84% reduction. The median reduction in IMR was 84% (range, 66–89%).

## 4. Discussion

### 4.1. Principal findings

This study confirms the core photobiological principle established in the 1990s: porfimer sodium-mediated photodynamic therapy (PDT) can potently attenuate neointimal hyperplasia following balloon injury [19]. We replicated Eton’s protocol to facilitate historical comparison; it is recommended that future studies employ intravenous administration [19]. Furthermore, using a rigorous paired-artery control design, we observed a consistent and significant reduction in the intima-media ratio (median 82%, range 66–89%) in the three animals that survived the protocol, aligning with the magnitude of the effect reported in early foundational work [19,20]. This reaffirms that reactive oxygen species (ROS)-mediated cytotoxicity selectively targets proliferating vascular smooth muscle cells (VSMCs) [10].

### 4.2. Methodological reappraisal: feasibility challenges as a primary outcome

However, the most salient and instructive finding of this pilot study was the profound feasibility barrier it encountered. A 70% overall attrition rate, comprising 40% intraoperative mortality, 10% late postoperative death, and 20% technical exclusions, transcends a mere limitation and represents a critical outcome. In translational research, feasibility encompasses procedural safety, animal welfare, and model sustainability [21]. The extreme attrition observed indicates that the bilateral femoral artery injury model, under the employed perioperative conditions, presents an unacceptable risk. This compels a shift in perspective: for a pilot feasibility study, a high failure rate in protocol completion is a central result that critically informs translational viability.

**Analysis of Attrition Drivers:** High mortality resulted from a confluence of model-specific challenges. Bilateral femoral artery injury carries the risk of profound acute limb ischemia, which can induce systemic inflammatory responses and remote organ injury, exacerbating physiological compromise [16,17]. This was compounded by the inherent sensitivity of rabbits to anesthesia and major vascular surgery. Our experience underscores a key translational research question: when does a model morbidity invalidate its predictive value?

**Reframing Limitations into Imperatives:** Consequently, high attrition should not be interpreted as a flaw negating the biological signal in survivors, but as a definitive feasibility result of mandating protocol redesign. Future investigations must prioritize establishing a survivable model, potentially through (1) optimized anesthesia and intensive perioperative monitoring, (2) unilateral injury or alternative vascular access sites to mitigate bilateral ischemic insult, and (3) consideration of more robust animal models (e.g., porcine) for complex procedures. This candid analysis transforms our study into a valuable case report on preclinical model development, emphasizing that establishing feasibility is a prerequisite for generating robust efficacy data.

### 4.3. Contextualizing the translational stall of vascular PDT

The promising biological effects observed in this study are consistent with the body of preclinical work from the 1990s that initially generated enthusiasm for vascular PDT [20–22]. Despite this, the approach has not progressed to clinical application for restenosis, creating a translational gap of more than two decades. Our study, with its stark feasibility challenges, provides a tangible microcosm to help contextualize this stagnation.

**The Feasibility “Valley of Death”:** One underappreciated factor is the translational “valley of death” created by persistent preclinical model challenges, as exemplified by our high-attrition study. Early promising results [19,20] were generated in models that may have harbored similar, albeit underreported, feasibility issues. When subsequent attempts to refine the therapy or scale it for clinical trials encounter these recurrent technical and mortality hurdles — increasing costs, raising ethical concerns, and complicating data interpretation — momentum can dissipate. Cheung et al.’s 2004 follow-up study, while showing long-term efficacy, also noted procedural complexities and the need for further optimization, subtly hinting at persistent translational barriers [23].

Intraoperative death was an unintended outcome; however, if the animals had shown persistent MAP < 30 mmHg or cardiac arrest, they would have been euthanized immediately. No animal met the humane endpoint criteria because death occurred acutely.

**Competition and Platform Challenges:** The timeline of vascular PDT’s development intersected with the revolutionary rise of drug-eluting stents (DES) and, later, drug-coated balloons (DCB) for coronary and peripheral diseases [24]. These device-based, single-procedure solutions offer compelling clinical efficacy and a more straightforward regulatory and clinical adoption pathway than the two-step process (photosensitizer administration followed by light delivery) and skin photosensitivity management required for PDT [8]. The remarkable success of DES/DCB likely absorbed most industrial and clinical research resources in the anti-restenosis field [24,25]. Furthermore, achieving uniform and reproducible light delivery in arteries presents substantial engineering challenges not faced by device-based drug delivery.

Despite these efficacy advantages, DES and DCB are not without limitations — including late thrombosis, paclitaxel-related mortality concerns (now largely resolved but historically impactful), and residual restenosis in complex lesions. However, compared to PDT, these technologies offer distinct translational advantages: a single-procedure workflow, no postoperative photosensitivity precautions, established reimbursement pathways, and extensive physician familiarity. PDT, by contrast, requires two separate encounters (photosensitizer administration 24–48 hours before light delivery), dedicated intravascular light-diffusing catheters, management of prolonged skin photosensitivity (4–6 weeks), and rigorous dosimetry to avoid vessel wall necrosis. These practical barriers — not merely biological efficacy — have historically limited clinical adoption and commercial investment, independent of the feasibility challenges documented in the present study. In summary, the translational stall of vascular PDT is likely multifactorial, resulting from (1) persistent preclinical model feasibility challenges that increase the risk and cost of development (as exemplified here), (2) the overwhelming clinical and commercial success of competing technologies (DES, DCB), and (3) the inherent complexities of the PDT platform itself for cardiovascular applications [8]. For vascular PDT to be successfully revisited, future research must explicitly address these historical roadblocks through the development of improved models, next-generation photosensitizers, and integrated light-delivery technologies.

### 4.4. Study strengths, limitations, and future directions

**Strengths:** The paired-artery control design was a critical methodological strength, controlling for inter-animal variability in pharmacokinetics and biological response and providing high internal validity for the observed biological effect, despite the small sample size.

**Limitations and Sample Size Considerations:** The final cohort of three animals (n = 3 per group) severely limited the generalizability of the results and precluded definitive conclusions. However, the paired design, consistent treatment direction across all subjects, large effect size (Cohen’s d = 2.8), and statistical significance (P < 0.001) support the validity of the observed biological signal as compelling pilot data. These findings must be interpreted as hypothesis-generating.

**Future Directions for Vascular PDT:** If the field is revisited, research must first address the feasibility challenges outlined above. Subsequent priorities include systematic PDT dose-optimization studies [10], mechanistic validation of ROS production and cell death pathways [3,10], and evaluation of combination strategies with current standards of care [25]. Long-term translation would require the development of dedicated intravascular light delivery catheters and safety studies using physiologically relevant large animal models.

### 4.5. Summary: Value of transparent historical reporting

We acknowledge the historical significance of this study. This publication is justified not by the novelty of the concept but by the transparent reporting of a complete dataset, including detailed dosimetry parameters (630 nm, ~ 5.6 J/cm²) and, crucially, a concrete and clear set of cautions for future investigators. At a time when peripheral arterial restenosis remains a significant clinical challenge [3,4], and interest in localized vascular therapies may resurge, this study serves as a valuable methodological case for future studies. It provides a foundational reference and a clear set of cautions to guide the design of more robust, ethical, and translatable studies.

## 5. Conclusion

This historical pilot study confirmed the potent biological effect of porfimer sodium-mediated photodynamic therapy (PDT) on neointimal hyperplasia in a rabbit model, consistent with foundational research from the 1990s [19,20]. Using a rigorous paired-artery design, a strong and consistent reduction in in intimal hyperplasia was observed in the surviving animals. However, the most significant contribution of this study lies in its transparent documentation and critical analysis of a profound feasibility barrier: an unsustainable 70% procedural attrition rate. This outcome shifts the primary value of the study from a simple efficacy report to a methodological case study that underscores the critical importance of model viability and procedural safety in translational research. A detailed analysis of the causes of attrition, including the challenges of bilateral femoral access [16,17] and perioperative management, provides a concrete “hazard map” for future studies.

The translational stall of vascular PDT over the past two decades can be partially contextualized by such persistent preclinical feasibility challenges, alongside the rise of competing technologies such as drug-eluting stents and drug-coated balloons [24,26]. To meaningfully revisit this field, future studies must first address these historical roadblocks by employing refined animal models, optimized supportive care, and potentially next-generation laser-related therapy and novel photosensitizers [25,27,28].

This reassessment serves as an academic analysis of translational research processes. It validates a known biological effect while extracting essential methodological lessons from the high-attrition pilot phase. By providing a complete dataset, candid feasibility assessment, and clear directives for protocol optimization, this study aims to inform and de-risk future efforts to explore localized photodynamic approaches for the prevention of vascular restenosis.

### 5.1. Disclosures of AI technologies in preparation of this manuscript

We used ChatGPT and Paperpal for typo detection and grammar polishing. The authors reviewed and edited the content as needed and took full responsibility for the article.

## Supporting information

S1 File Raw database summary. Complete raw dataset of intima-media ratio (IMR) measurements for all arteries included in the final analysis (Rabbits C, D, and E), including PDT-treated and contralateral control arteries. Data are provided in English and include all morphometric measurements used for statistical analysis.
(XLSX)

S2 File Survival table. Structured summary of animal survival outcomes, including intima-media ratio data stratified by animal and by treatment group, corresponding to the data presented in Table 2.
(DOCX)

S1 Fig Microscopic slide of animal C demonstrates obvious intimal hyperplasia after angioplasty injury without photodynamic therapy. (PNG)

S2 Fig Microscopic slide of animal C demonstrates inhibition of intimal hyperplasia after angioplasty injury after photodynamic therapy. (PNG)

S3 Fig Microscopic slide of animal C demonstrates inhibition of intimal hyperplasia after angioplasty injury after photodynamic therapy. (PNG)

S4 Fig Microscopic slide of animal D demonstrates obvious intimal hyperplasia after angioplasty injury without photodynamic therapy. (PNG)

S2 Table Animal Attrition. (DOCX)
