# Supplementary material for: Reappraisal of a historical porfimer sodium photodynamic therapy study for vascular restenosis: Efficacy, high procedural mortality, and methodological insights from a rabbit balloon-injury model
Source: PLoS One. 2026 Jun 22;21(6):e0350675. doi: 10.1371/journal.pone.0350675 (PMC13286160; doi:10.1371/journal.pone.0350675)
Supplement: S2 File — Structured summary of animal survival outcomes, including intima-media ratio data stratified by animal and by treatment group, corresponding to the data presented in Table 2. (DOCX) [file pone.0350675.s002.docx]

**Table 1. Intima–Media Ratio (IMR, pixels)**

1. **– By PDT Group**

| *Group (PDT)* | *IMR (mean ± SD, pixels)** |
| --- | --- |
| *PDT = 1 (Photodynamic Therapy)* | 0.291 ± 0.072* |
| *PDT = 0 (Control)* | 1.647 ± 0.554* |

* Exact p-value = 0.0004038 by paired Wilcoxon signed-rank test

1. **– By Animal**

| *Animal* | *IMR (mean ± SD, pixels)#* |
| --- | --- |
| *C* | 1.288 ± 1.142 |
| *D* | 0.812 ± 0.576 |
| *E* | 0.808 ± 0.581 |

1. #Kruskal-Wallis chi-squared = 0.44289, df = 2, p-value = 0.8014
