# Supplementary figures and images for: Reappraisal of a historical porfimer sodium photodynamic therapy study for vascular restenosis: Efficacy, high procedural mortality, and methodological insights from a rabbit balloon-injury model
Source: PLoS One. 2026 Jun 22;21(6):e0350675. doi: 10.1371/journal.pone.0350675 (PMC13286160; doi:10.1371/journal.pone.0350675)

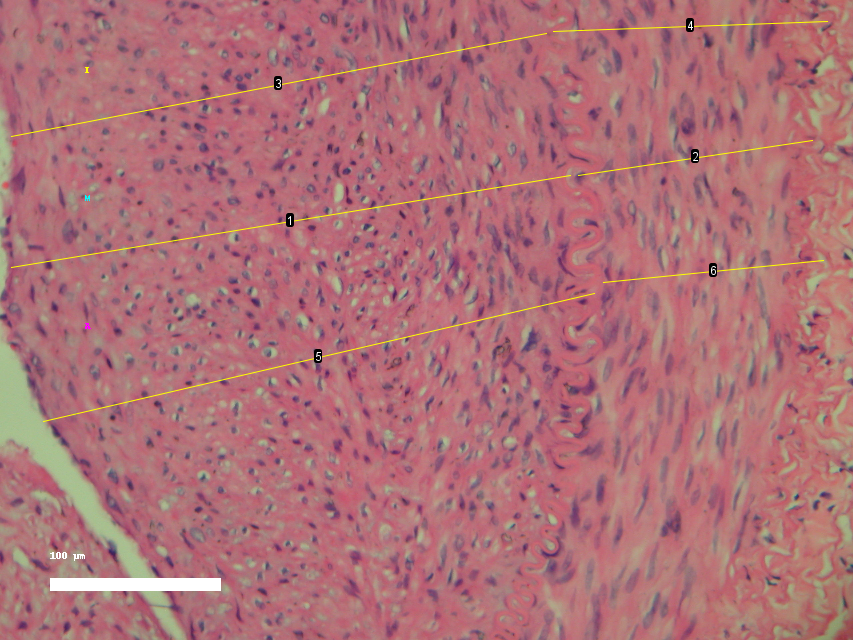

Supplement: S1 Fig — (PNG) [file pone.0350675.s003.png]

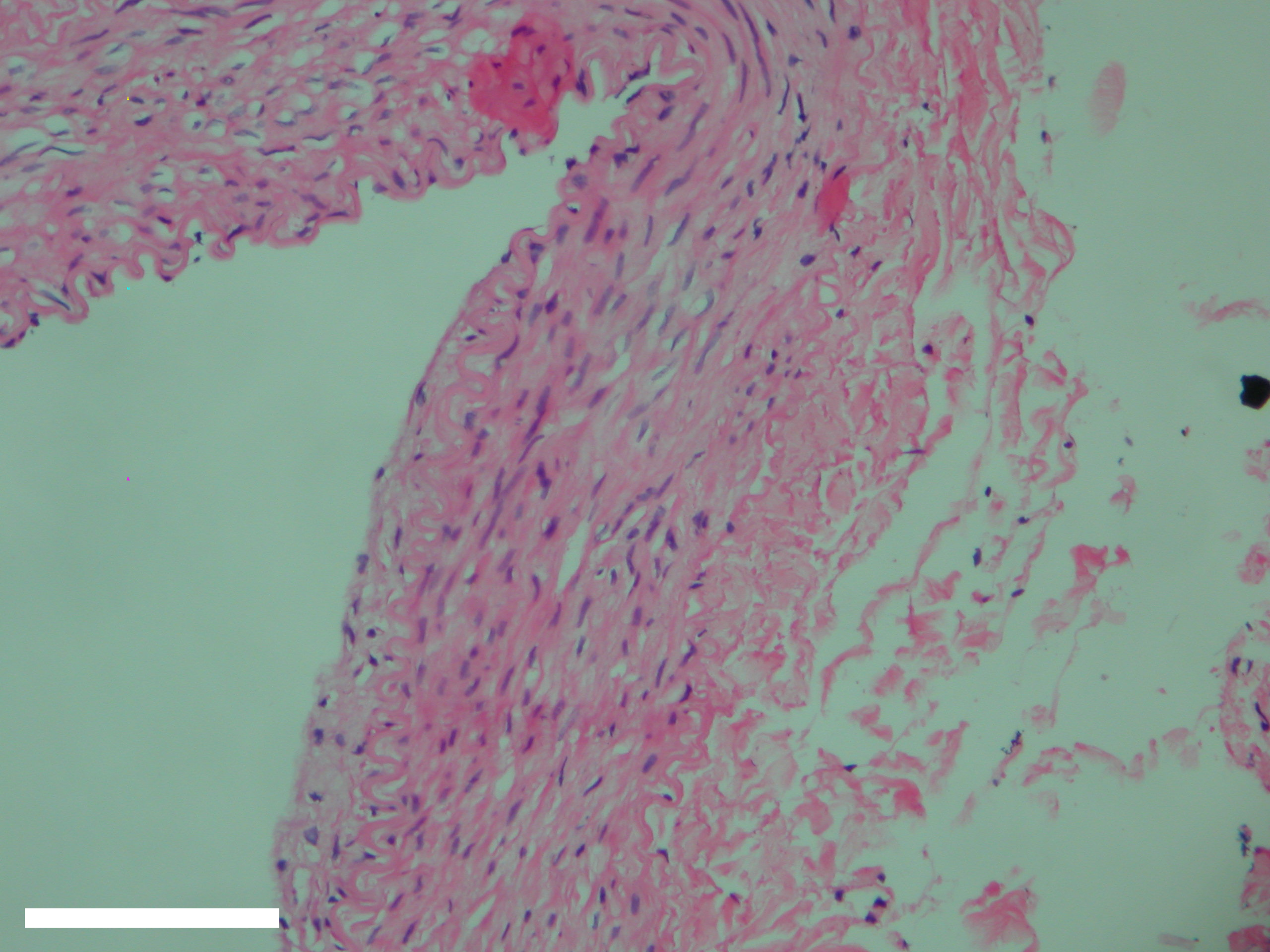

Supplement: S2 Fig — (PNG) [file pone.0350675.s004.png]

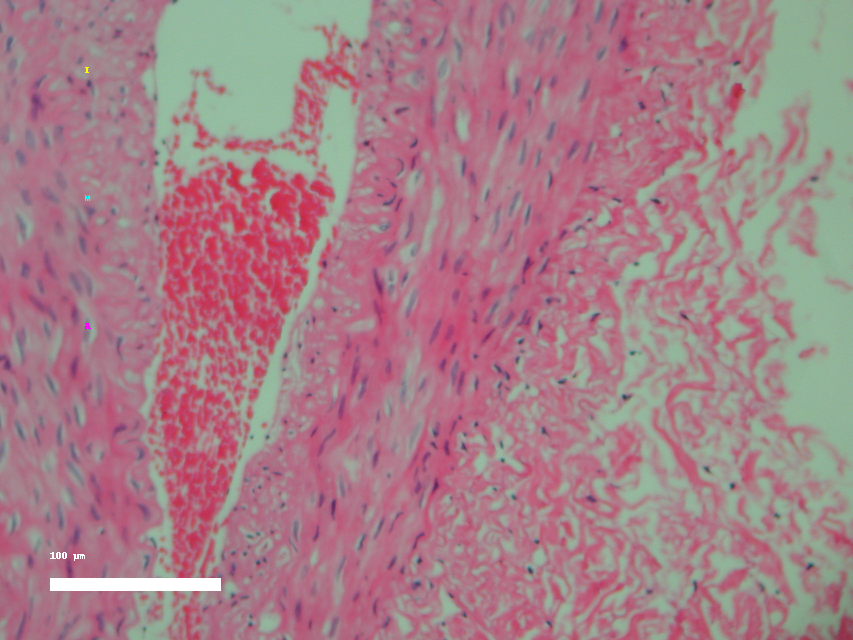

Supplement: S3 Fig — (PNG) [file pone.0350675.s005.png]

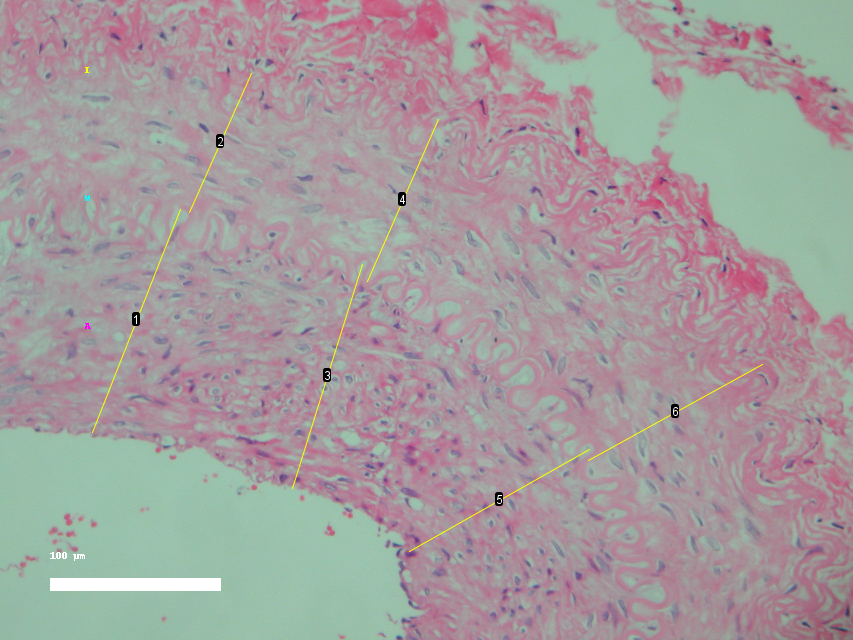

Supplement: S4 Fig — (PNG) [file pone.0350675.s006.png]
