# Supplementary material for: Reappraisal of a historical porfimer sodium photodynamic therapy study for vascular restenosis: Efficacy, high procedural mortality, and methodological insights from a rabbit balloon-injury model
Source: PLoS One. 2026 Jun 22;21(6):e0350675. doi: 10.1371/journal.pone.0350675 (PMC13286160; doi:10.1371/journal.pone.0350675)
Supplement: S2 Table — (DOCX) [file pone.0350675.s007.docx]

**Table 2. Animal Attrition and Procedural Outcome Summary**

| **Animal** | **Fate** | **Timing of Event** | **Suspected Cause of Death / Exclusion** | **PDT Delivered** | **Contributing Procedural Factors** | **Post-mortem Data Available** | **Outcome / Notes** |
| --- | --- | --- | --- | --- | --- | --- | --- |
| A | Intraoperative death | During surgery | Suspected anesthesia-related cardiorespiratory failure | No | Bilateral femoral exposure; no capnography/hemodynamic monitoring | No | Excluded — no tissue available |
| B | Intraoperative death | During surgery | Suspected anesthesia-related cardiorespiratory failure | No | Bilateral femoral exposure; no capnography/hemodynamic monitoring | No | Excluded — no tissue available |
| C | Survived to endpoint | Day 21 | N/A — survived | Yes | Bilateral femoral exposure | No* | IMR: Control 2.31 → PDT 0.25 (89% reduction); included in final analysis |
| D | Survived to endpoint | Day 21 | N/A — survived | Yes | Bilateral femoral exposure | No* | IMR: Control 1.19 → PDT 0.40 (66% reduction); included in final analysis |
| E | Survived to endpoint | Day 21 | N/A — survived | Yes | Bilateral femoral exposure | No* | IMR: Control 1.44 → PDT 0.23 (84% reduction); included in final analysis |
| F | Intraoperative death | During surgery | Suspected anesthesia-related cardiorespiratory failure | No | Bilateral femoral exposure; no capnography/hemodynamic monitoring | No | Excluded — no tissue available |
| G | Intraoperative death | During surgery | Suspected anesthesia-related cardiorespiratory failure | No | Bilateral femoral exposure; no capnography/hemodynamic monitoring | No | Excluded — no tissue available |
| H | Survived; technically excluded | Post-op (before Day 21) | Distal occlusion of target vessel | Yes | Bilateral femoral exposure; likely thrombosis at repair site | No | Excluded — vessel not analyzable |
| I | Survived; technically excluded | Post-op (before Day 21) | Distal occlusion of target vessel | Yes | Bilateral femoral exposure; likely thrombosis at repair site | No | Excluded — vessel not analyzable |
| J | Late postoperative death | Week 3 | Suspected wound infection / sepsis | Yes | Bilateral femoral wound; inadequate postoperative surveillance | No | Excluded — died before tissue harvest |

**Notes:**

Animal labeling (A–J) is assigned sequentially for this table. Original study records used animals identified as Rabbits A–J, with Rabbits C, D, and E corresponding to the three analyzable survivors.

PDT = photodynamic therapy; IMR = intima–media ratio; N/A = not applicable.

* Formal gross post-mortem organ evaluation (liver, lungs, kidneys, heart) was not performed on surviving animals at the study endpoint, representing a limitation of the original protocol. Routine histological assessment was limited to the femoral artery specimens.

† All four intraoperative deaths occurred prior to PDT delivery, indicating that procedural mortality was attributable to bilateral balloon injury and anesthesia burden rather than the PDT protocol itself.

Overall attrition: 4/10 intraoperative deaths (40%); 1/10 late postoperative death (10%); 2/10 technical exclusions (20%); 3/10 completed analysis (30%).
