# Supplementary material for: Reappraisal of a historical porfimer sodium photodynamic therapy study for vascular restenosis: Efficacy, high procedural mortality, and methodological insights from a rabbit balloon-injury model
Source: PLoS One. 2026 Jun 22;21(6):e0350675. doi: 10.1371/journal.pone.0350675 (PMC13286160; doi:10.1371/journal.pone.0350675)
Supplement: Supplementary file 4 [file pone.0350675.s009.docx]

**Manuscript Number: PONE-D-25-68528**

**Title:** Reappraisal of a historical porfimer sodium photodynamic therapy study for vascular restenosis: Efficacy, high procedural mortality, and methodological insights from a rabbit balloon-injury model

**Journal:** PLOS ONE

**Date:** March 2, 2026

Dear Dr. Sojitra and Reviewers,

We sincerely thank the Academic Editor and both Reviewers for their thorough and constructive evaluation of our manuscript. Their comments have substantially strengthened the work. We have addressed every point raised and detailed our responses below. All changes are highlighted in the revised manuscript with tracked changes.

For clarity, each original comment is reproduced in italics, followed by our response and the specific changes made to the manuscript.

# **RESPONSES TO ACADEMIC EDITOR COMMENTS**

## **Editor Comment 1: Scientific acceptance criteria and model justification**

*The manuscript presented very old data and over 20 years many scientific acceptance criteria have been changed particularly, justification of animal model and why safety response data should be considered relevant to human, and response of femoral to coronary arteries.*

Response: We appreciate this important point. We have expanded Section 4.3 (Contextualizing the Translational Stall) and added a new paragraph in the Discussion to directly address the evolution of scientific standards over the past two decades. We now explicitly discuss (1) how current ARRIVE 2.0 guidelines and 3Rs principles reframe this historical dataset's significance, (2) the acknowledged translational gap between rabbit femoral and human peripheral (not coronary) arteries in terms of vessel diameter, wall composition, and biomechanical environment, and (3) why the documented safety/feasibility failures are arguably more relevant to modern translational science than efficacy endpoints alone. We also clarify throughout that comparisons are made to peripheral (femoral/femoropopliteal) arteries, not coronary arteries, consistent with our stated clinical target. The following sentence has been added to Section 4.3:

Added text (Section 4.3): "It is important to note that the rabbit femoral artery model, while a standard in preclinical vascular biology, carries acknowledged translational limitations relative to human peripheral arteries — including differences in vessel diameter, wall architecture, and susceptibility to inflammatory injury. These differences, in addition to the feasibility failures documented here, must be explicitly acknowledged when interpreting the translational relevance of historical porfimer sodium PDT data. The current report does not claim direct clinical extrapolation, but rather offers methodological lessons for future preclinical studies designed with modern standards."

## **Editor Comment 2: Unexplained mortality and gross post-mortem evaluation**

*Furthermore, mortality rate has been higher which remains unexplained. Gross evaluation on non-treated organs and post mortem details would be necessary to appraise those data for readers to repeat experiments without additional animal harm.*

Response: We fully agree that this is a critical gap in the original data. We acknowledge candidly that formal post-mortem organ examination (gross pathology of liver, kidneys, lungs, and heart) was not performed on the animals that died intraoperatively or postoperatively — a significant limitation of this historical pilot study. We have added an explicit statement in Section 2.2 (Surgical Procedure) and Section 3.1 (Animal Survival) acknowledging this deficiency and its implications. We have also added a new Table (Table 2: Animal Attrition Summary) in the Results section that provides a structured breakdown of each animal's fate, timing of death, suspected cause, and whether post-mortem data are available.

Added text (Section 3.1): "Formal gross post-mortem organ evaluation was not performed on animals that died intraoperatively or in the early postoperative period — a significant limitation of this historical pilot study. The absence of systematic organ-level data prevents definitive attribution of death to specific causes (e.g., systemic ischemia-reperfusion injury, anesthesia-related cardiac/respiratory failure, or hemorrhage). Future investigators should include structured post-mortem protocols as a mandatory component of any bilateral femoral injury model to enable complete cause-of-death attribution and to inform humane endpoint criteria. This limitation is explicitly noted in Table 2."

## **Editor Comment 3: Anesthesia protocol explanation**

*The explanation states that high mortality likely resulted from a confluence of model-specific challenges. Animal death suspected due to anesthesia-related complications is hard to understand, because your lab must have internal protocol anesthesia dose.*

Response: We thank the editor for this important challenge. The anesthesia protocol used (intramuscular xylazine 5 mg/kg + ketamine 35 mg/kg, with supplemental half-dose if movement observed) was indeed our institutional protocol at the time (early 2003). However, this combination carries well-documented risks in rabbits, including respiratory depression, cardiovascular suppression, and dose variability due to intramuscular absorption. Critically, our protocol lacked intra-arterial pressure monitoring and capnography — monitoring tools that are now considered standard for major vascular procedures in rabbits. We have substantially revised Section 2.2 to be more explicit about the anesthesia protocol's specific limitations, and Section 4.2 now includes a more nuanced mechanistic discussion of why even a standard-dose protocol may have resulted in high mortality under these specific procedural conditions (bilateral femoral clamping, prolonged surgery, volume shifts).

Revised text (Section 2.2): "General anesthesia was induced using intramuscular xylazine hydrochloride (5 mg/kg) and ketamine hydrochloride (35 mg/kg), which represented the institutional rabbit anesthesia protocol at the time of study (2003). Anesthetic depth was assessed by loss of pedal withdrawal reflex and maintained with a supplemental half-dose upon observed movement. Critically, continuous intra-arterial pressure monitoring and capnography were not employed. These monitoring modalities are now recognized as essential for detecting early cardiovascular compromise during major vascular procedures in rabbits, and their absence represents a significant limitation of the original protocol that likely contributed to the high intraoperative mortality. Body temperature was maintained using a homeothermic blanket. Postoperative analgesia was provided with buprenorphine (0.03 mg/kg, IM) as per institutional guidelines."

## **Editor Comment 4: Crossover data to add scientific merit**

*To be more scientific contents, we highly recommend to present any crossover data which can add scientific merit to contents. This data may significantly contribute to final decision.*

Response: We interpret "crossover data" as referring to any additional comparative or paired data that could strengthen the biological conclusions. In our within-subject paired design, each animal inherently served as its own crossover control (PDT-treated right/left artery vs. untreated contralateral artery). We have made this paired/crossover structure more explicit in the Methods (Section 2.3) and Results (Section 3.2), including a new supplementary figure (S4 Figure) showing individual animal paired IMR comparisons side-by-side with connecting lines to visualize the within-animal treatment effect. This paired visualization approach — analogous to a crossover display — directly illustrates the consistency of the PDT effect across all three surviving animals and strengthens the biological interpretation. We have also added the individual raw measurement data to the Zenodo repository (updated S2 file).

## **Editor Comment 5: Histology slides insufficient — full artery views required**

*Histology slides are insufficient and you should provide full view of artery for all cases (C, D, E and Control).*

Response: We fully accept this important request. We have prepared additional high-resolution full cross-sectional H&E images (low-magnification, 4x objective, full vessel lumen view) for all six analyzed artery sections (Animals C, D, and E — both PDT-treated and control sides). These have been added as a new Supplementary Figure (S4: Complete histological panel for all analyzed animals). The original Figure 1 (representative Animal E images at higher magnification with measurement overlay) is retained in the main text. The caption for Figure 1 has been updated to direct readers to the comprehensive S4 panel.

## **Editor Comment 6: "rats" error in Line 119**

*Line 119, mentioned rats, you should correct it.*

Response: Corrected. Line 119 (Section 2.3) has been changed from "A 425-µm optical fiber with a cylindrical diffuser tip (25 mm) was inserted into the target artery of the rats" to "...the target artery of the rabbits." We apologize for this typographical error.

# **RESPONSES TO PLOS ONE JOURNAL REQUIREMENTS**

## **Journal Requirement 1: PLOS ONE style requirements**

Response: The revised manuscript has been formatted to comply with PLOS ONE style templates. Line numbers have been added throughout, figure file names have been revised to comply with naming conventions, and the title page has been reformatted per the PLOS ONE title/author/affiliation template.

## **Journal Requirement 2: Animal methods — sacrifice and suffering alleviation**

Response: We have expanded the Methods section to include these details. Added to Section 2.2: "Postoperative pain management included buprenorphine (0.03 mg/kg, IM, every 8–12 hours for 48 hours). Animals were monitored twice daily for signs of distress, wound dehiscence, or limb ischemia. Humane endpoints included signs of severe respiratory distress, sustained limb necrosis, or inability to ambulate and access food/water." Added to Section 2.4: "At the 21-day study endpoint, euthanasia was performed by intravenous pentobarbital overdose (100 mg/kg, via marginal ear vein) under deep anesthesia (xylazine/ketamine as described above), consistent with institutional guidelines and the AVMA Guidelines for the Euthanasia of Animals."

## **Journal Requirement 3: Funder role statement**

Response: The funding statement has been updated to include the funder's role. Revised statement: "The Far Eastern Memorial Hospital Research Fund (FEMH 92-D-008) provided financial and administrative support for this study. The funders had no role in study design, data collection and analysis, decision to publish, or preparation of the manuscript." This statement appears in both the manuscript and is provided in this cover letter for the submission form update.

## **Journal Requirement 4: Full ethics statement in Methods**

Response: The full ethics statement has been incorporated into Section 2.1 of the Methods. Revised text: "All procedures were approved by the Institutional Animal Care and Use Committee (IACUC) of Far Eastern Memorial Hospital, New Taipei, Taiwan (Protocol Number: FEMH-92-D-008, approved March 15, 2003). This study was conducted in strict accordance with the recommendations of the Guide for the Care and Use of Laboratory Animals (NIH) and the Taiwan Animal Protection Act. As this study involved no human participants, no human consent was required. All animal surgeries were performed under general anesthesia, and all efforts were made to minimize suffering, as described in Sections 2.2 and 2.4."

## **Journal Requirement 5: Data availability plan**

Response: All raw data are currently deposited and publicly available at Zenodo (https://doi.org/10.5281/zenodo.18054401). The Zenodo repository has been updated with the complete individual animal measurement dataset. The data availability statement in the manuscript has been updated to confirm: "All relevant data are within the manuscript and its Supporting Information files, and the complete raw dataset is publicly available at Zenodo (https://doi.org/10.5281/zenodo.18054401)."

# **RESPONSES TO REVIEWER #1 COMMENTS**

## **Reviewer #1 Comment 1: Sample size and limited novelty**

*The manuscript is a good study revisiting the PDT therapy for vascular restenosis however the sample size is very low to make a conclusion. There is also not much novelty in the study as this was a study that was performed a while ago and published. There is great potential in exploring the PDT therapy for vascular restenosis however additional pre-clinical data is required with an increased sample size and the use of a different animal model as suggested by the authors in the manuscript. It is unfortunate that there was 40% mortality rate and 70% of the data were unusable.*

Response: We thank Reviewer #1 for this balanced assessment. We agree entirely with the characterization of the sample size limitations and the lack of conceptual novelty — both are acknowledged explicitly in the manuscript as core features of the work rather than flaws to be minimized. The manuscript's primary contribution is not a novel efficacy claim but a transparent feasibility and methodological case study — a genre of scientific reporting increasingly recognized as valuable for the translational research community (per PLOS ONE's own scope statement on "negative and null results"). We have strengthened the framing in the Abstract and Introduction to make this distinction even clearer, so readers immediately understand the study's contribution type.

Specifically, we have revised the Abstract Conclusions to lead with the methodological/feasibility contribution: "This pilot reappraisal does not claim conceptual novelty but provides the scientific community with a complete, transparent historical dataset including documented PDT parameters, a candid feasibility analysis revealing critical translational barriers, and concrete protocol recommendations to guide future investigators — without necessitating additional animal studies."

We also agree with Reviewer #1's recommendation for future studies using larger sample sizes and alternative animal models, and this is now more prominently stated in Section 4.4 (Future Directions), which now explicitly recommends the porcine model as the preferred next step.

# **RESPONSES TO REVIEWER #2 COMMENTS (Dr. Bhavdip Doshi)**

## **Reviewer #2 Comment 1: Quantitative comparison with recent literature and alternative therapies**

*While the historical context is well established, the manuscript should provide a direct quantitative comparison of efficacy and mortality outcomes with more recent vascular PDT literature and alternative therapies (e.g., current drug-eluting stent (DES), drug coated balloon DCB restenosis rates & IMR).*

Response: We have added a new paragraph in Section 4.3 providing quantitative contextual comparison. Key additions: Contemporary drug-coated balloon (DCB) therapy for femoropopliteal disease (IN.PACT SFA trial, Tepe et al., 2015, reference [24]) shows 12-month primary patency of 82.2% vs. 52.4% for standard PTA. Drug-eluting stents for infrapopliteal disease show restenosis rates of approximately 20–30% at 12 months [Lim et al., 2024, reference [4]]. By contrast, our PDT-treated arteries (in a rabbit model, not directly comparable) showed 82% median IMR reduction at 21 days — a strong biological signal but one that cannot be directly translated to clinical patency rates without further development. This contextualizes the biological promise while maintaining scientific accuracy about the translational gap.

## **Reviewer #2 Comment 2: Breakdown of causes of death and correlation with procedural parameters**

*The authors report a high procedural mortality, but further breakdown is needed. Please include a table or figure summarising causes of death and the distribution (e.g., reasoning for intraoperative & postoperative) and clarify if mortality correlates with specific, negligence of using capnography, PDT parameters (dose, light exposure/distribution, control of laser power, excessive balloon injury, excessive irradiation time, etc.)*

Response: We have added Table 2 (Animal Attrition and Cause-of-Death Summary) to the Results section, presenting each of the 10 animals with: enrollment number, fate (survived/died), timing of death, primary suspected cause, whether PDT was delivered, and contributing procedural factors. Regarding correlation with PDT parameters specifically: PDT was delivered only to the surviving animals (C, D, E) after both arteries had been injured. The four animals that died intraoperatively all died prior to or during the PDT step, suggesting that mortality was primarily attributable to the bilateral balloon injury and anesthesia burden, not to the PDT itself. This important point is now explicitly stated in Section 3.1 and discussed in Section 4.2. The absence of capnography and continuous hemodynamic monitoring is now explicitly identified as a specific correctable contributor to mortality in Section 4.2.

## **Reviewer #2 Comment 3: Sample size justification and confounders in statistical section**

*The statistical analysis section could be expanded to include additional details regarding sample size justification and methods used to address potential confounders.*

Response: We have expanded Section 2.5 to include: (1) A formal sample size rationale: as a feasibility pilot, formal a priori power calculation was not the primary driver; however, the paired design with a detected Cohen's d of 2.8 exceeds the threshold for detecting large effects (d > 1.5) even with n = 3 pairs, providing adequate evidence of biological signal as pilot data. (2) A discussion of confounders addressed by design: the within-subject paired design controls for inter-animal pharmacokinetic variability, systemic inflammatory responses, and individual differences in neointimal response magnitude. (3) A statement of residual confounders: the small surviving cohort may represent a survival bias (i.e., animals that survived may share characteristics making them more resilient, potentially influencing baseline arterial biology), which is now explicitly discussed as a limitation.

## **Reviewer #2 Comment 4: Ethical implications of high-mortality preclinical studies**

*Consider including a brief discussion on the ethical implications of high-mortality preclinical studies and how modern standards might address these concerns.*

Response: We have added a dedicated paragraph in Section 4.2 titled "Ethical Reappraisal Under Modern Standards." This paragraph discusses: (1) the 3Rs framework (Replacement, Reduction, Refinement) and how this historical protocol would require substantial modification under current ethical standards; (2) the argument that transparent publication of high-attrition pilot data serves an ethical function by enabling future investigators to avoid repeating failed protocols; and (3) how modern pre-approval requirements (e.g., harm-benefit analysis, humane endpoint definitions, mandatory pilot feasibility assessment) would likely prevent the bilateral injury protocol from being approved as originally designed.

# **SUMMARY OF CHANGES TO THE MANUSCRIPT**

The following table summarizes all major revisions:

**1. Line 119:** Corrected "rats" to "rabbits" (typographical error).

**2. Section 2.1 (Ethics statement):** Full IACUC statement with protocol number, approval date, and compliance with NIH Guide now included in Methods.

**3. Section 2.2 (Anesthesia):** Expanded to include postoperative analgesia (buprenorphine), homeothermic blanket use, and explicit statement that capnography/arterial monitoring were absent and why this likely contributed to mortality.

**4. Section 2.4 (Euthanasia):** Method of euthanasia (IV pentobarbital overdose) now explicitly stated.

**5. Section 2.5 (Statistics):** Sample size rationale, confounders addressed by design, and survival bias limitation added.

**6. Section 3.1 (Survival results):** Added Table 2 (attrition summary), explicit statement about absence of post-mortem organ data, and clarification that PDT-related mortality was not attributable to PDT parameters.

**7. Section 4.2 (Discussion — feasibility):** Expanded with more specific anesthesia failure analysis, new "Ethical Reappraisal" paragraph, and correctable vs. non-correctable attrition drivers.

**8. Section 4.3 (Translational stall):** Added quantitative DES/DCB comparison, explicit statement on rabbit-to-human translational limitations, and expanded 3Rs ethical discussion.

**9. Section 4.4 (Future directions):** Porcine model explicitly recommended as preferred next step.

**10. Supplementary S4:** Full cross-sectional H&E images for all 6 analyzed arteries (Animals C, D, E — both sides) added.

**11. Funder role statement:** Updated to confirm funders had no role in study design, data collection, analysis, or publication.

**12. Data availability:** Zenodo repository confirmed as publicly accessible; individual raw measurement data added.

We believe these revisions substantially address all concerns raised and are confident the revised manuscript now meets PLOS ONE's publication standards for a transparent methodological pilot study.

We sincerely appreciate the editorial team's time and consideration, and we welcome any further feedback.

Sincerely,

**_______________________**

**Ai-Hsien Adams Li, MD, PhD, MPH**

Corresponding Author

Cardiovascular Center, Far Eastern Memorial Hospital

New Taipei, Taiwan

las1012.tw@gmail.com
