# Supplementary material for: Reappraisal of a historical porfimer sodium photodynamic therapy study for vascular restenosis: Efficacy, high procedural mortality, and methodological insights from a rabbit balloon-injury model
Source: PLoS One. 2026 Jun 22;21(6):e0350675. doi: 10.1371/journal.pone.0350675 (PMC13286160; doi:10.1371/journal.pone.0350675)
Supplement: Supplementary file 5 [file pone.0350675.s010.docx]

**Response to Reviewers — Revision R2**

**Manuscript:** PONE-D-25-68528R1
**Title:** Reappraisal of a historical porfimer sodium photodynamic therapy study for vascular restenosis: Efficacy, high procedural mortality, and methodological insights from a rabbit balloon-injury model
**Journal:** PLOS ONE

Dear Dr. Sojitra and Reviewers,

We thank the Academic Editor and both Reviewers for their positive assessment and for the final, minor suggestions to strengthen the manuscript. We have addressed the remaining point raised by Reviewer #2 and have performed a thorough language and formatting polish.

Below is our point‑by‑point response.

**Reviewer #2 Comment (remaining suggestion):**

*The authors have added quantitative comparisons with drug-eluting stents (DES) and drug-coated balloons (DCB), but the discussion could be expanded to include the limitations of PDT compared to these technologies, particularly in terms of clinical adoption and cost*‑*effectiveness.*

**Response:** We agree that this adds important balance to the translational discussion. We have expanded Section 4.3 with a new paragraph that explicitly compares PDT’s practical limitations (two‑step procedure, skin photosensitivity, specialized light‑delivery equipment, dosimetry challenges) against the clinical‑adoption advantages of DES and DCB (single procedure, no photosensitivity, established reimbursement). This contextualizes why PDT has struggled to compete commercially despite biological efficacy. The new text has been incorporated into Section 4.3 of the revised manuscript.

**Additional revisions:**

- The entire manuscript has been proofread for minor typographical and grammatical errors.
- Formatting has been checked for consistency with PLOS ONE guidelines (figure labels, reference style, spacing).
- No changes have been made to the data, results, or conclusions.

We believe the manuscript is now ready for formal acceptance. Thank you again for the constructive and efficient review process.

Sincerely,

__________________________

Ai‑Hsien Adams Li, MD, PhD, MPH
Corresponding Author
