# Supplementary material for: Reappraisal of a historical porfimer sodium photodynamic therapy study for vascular restenosis: Efficacy, high procedural mortality, and methodological insights from a rabbit balloon-injury model
Source: PLoS One. 2026 Jun 22;21(6):e0350675. doi: 10.1371/journal.pone.0350675 (PMC13286160; doi:10.1371/journal.pone.0350675)
Supplement: Peer review history

# Peer review: Decision Letter 0

**DOI:** 10.1371/journal.pone.0350675.r001

**Authors:** Prakash Sojitra

22 Feb 2026

PONE-D-25-68528

Reappraisal of a historical porfimer sodium photodynamic therapy study for vascular restenosis: Efficacy, high procedural mortality, and methodological insights from a rabbit balloon-injury model

PLOS One

Dear Dr. Li,

Thank you for submitting your manuscript to PLOS ONE. After careful consideration, we feel that it has merit but does not fully meet PLOS ONE’s publication criteria as it currently stands. Therefore, we invite you to submit a revised version of the manuscript that addresses the points raised during the review process.

The manuscript presented very old data and over 20 years many scientific acceptance criteria have been changed particularly, justification of animal model and why safety repose data should be considered to human to specific model presented herein and response of femoral to coronary arteries.

Furthermore, mortality rate has been higher which remains unexplained. Gross evaluation on non-treated organs and post mortem details would be necessary to appraise those data for readers to repeat experiments without additional animal harm.

The explanation states that high mortality likely resulted from a confluence of model-specific challenges. Animal death suspected due to anesthesia-related complications is hard to understand, because your lab must have internal protocol anesthesia dose.

To be more scientific contents, we highly recommend to present any crossover data which can add scientific merit to contents. This data any significantly contribute final decision.

Histology slides are insufficient and you should provide full view of artery for all cases (C, D, E and Control)

Line 119, mentioned rats, you should correct it.

Please submit your revised manuscript by Apr 08 2026 11:59PM If you will need more time than this to complete your revisions, please reply to this message or contact the journal office at plosone@plos.org. When you're ready to submit your revision, log on to https://www.editorialmanager.com/pone/ and select the 'Submissions Needing Revision' folder to locate your manuscript file.

Please include the following items when submitting your revised manuscript:

- A letter that responds to each point raised by the academic editor and reviewer(s). You should upload this letter as a separate file labeled 'Response to Reviewers'.
- A marked-up copy of your manuscript that highlights changes made to the original version. You should upload this as a separate file labeled 'Revised Manuscript with Track Changes'.
- An unmarked version of your revised paper without tracked changes. You should upload this as a separate file labeled 'Manuscript'.

If you would like to make changes to your financial disclosure, please include your updated statement in your cover letter. Guidelines for resubmitting your figure files are available below the reviewer comments at the end of this letter.

If applicable, we recommend that you deposit your laboratory protocols in protocols.io to enhance the reproducibility of your results. Protocols.io assigns your protocol its own identifier (DOI) so that it can be cited independently in the future. For instructions see: https://journals.plos.org/plosone/s/submission-guidelines#loc-laboratory-protocols. Additionally, PLOS ONE offers an option for publishing peer-reviewed Lab Protocol articles, which describe protocols hosted on protocols.io. Read more information on sharing protocols at https://plos.org/protocols?utm_medium=editorial-email&utm_source=authorletters&utm_campaign=protocols.

We look forward to receiving your revised manuscript.

Kind regards,

Prakash Sojitra, PhD

Academic Editor

PLOS One

Journal Requirements:

When submitting your revision, we need you to address these additional requirements.

1. Please ensure that your manuscript meets PLOS ONE's style requirements, including those for file naming. The PLOS ONE style templates can be found at

https://journals.plos.org/plosone/s/file?id=wjVg/PLOSOne_formatting_sample_main_body.pdf and

https://journals.plos.org/plosone/s/file?id=ba62/PLOSOne_formatting_sample_title_authors_affiliations.pdf

2. To comply with PLOS One submissions requirements, in your Methods section, please provide additional information regarding the experiments involving animals and ensure you have included details on (1) methods of sacrifice, (2) efforts to alleviate suffering.

3. Thank you for stating the following financial disclosure:

“The Far Eastern Memorial Hospital Research Fund provided financial and administrative support for this study.”

Please state what role the funders took in the study.  If the funders had no role, please state: "The funders had no role in study design, data collection and analysis, decision to publish, or preparation of the manuscript."

If this statement is not correct you must amend it as needed.

Please include this amended Role of Funder statement in your cover letter; we will change the online submission form on your behalf.

4. Please include your full ethics statement in the ‘Methods’ section of your manuscript file. In your statement, please include the full name of the IRB or ethics committee who approved or waived your study, as well as whether or not you obtained informed written or verbal consent. If consent was waived for your study, please include this information in your statement as well.

5. When completing the data availability statement of the submission form, you indicated that you will make your data available on acceptance. We strongly recommend all authors decide on a data sharing plan before acceptance, as the process can be lengthy and hold up publication timelines. Please note that, though access restrictions are acceptable now, your entire data will need to be made freely accessible if your manuscript is accepted for publication. This policy applies to all data except where public deposition would breach compliance with the protocol approved by your research ethics board. If you are unable to adhere to our open data policy, please kindly revise your statement to explain your reasoning and we will seek the editor's input on an exemption. Please be assured that, once you have provided your new statement, the assessment of your exemption will not hold up the peer review process.

Reviewer's Responses to Questions

**Comments to the Author**

1. Is the manuscript technically sound, and do the data support the conclusions?

The manuscript must describe a technically sound piece of scientific research with data that supports the conclusions. Experiments must have been conducted rigorously, with appropriate controls, replication, and sample sizes. The conclusions must be drawn appropriately based on the data presented.

Reviewer #1: Partly

Reviewer #2: Partly

**********

2. Has the statistical analysis been performed appropriately and rigorously?

Reviewer #1: Yes

Reviewer #2: Yes

**********

3. Have the authors made all data underlying the findings in their manuscript fully available?

The PLOS Data policy requires authors to make all data underlying the findings described in their manuscript fully available without restriction, with rare exception (please refer to the Data Availability Statement in the manuscript PDF file). The data should be provided as part of the manuscript or its supporting information, or deposited to a public repository. For example, in addition to summary statistics, the data points behind means, medians and variance measures should be available. If there are restrictions on publicly sharing data—e.g. participant privacy or use of data from a third party—those must be specified.

Reviewer #1: Yes

Reviewer #2: Yes

**********

4. Is the manuscript presented in an intelligible fashion and written in standard English?

PLOS ONE does not copyedit accepted manuscripts, so the language in submitted articles must be clear, correct, and unambiguous. Any typographical or grammatical errors should be corrected at revision, so please note any specific errors here.

Reviewer #1: Yes

Reviewer #2: Yes

**********

5. Review Comments to the Author

Please use the space provided to explain your answers to the questions above. You may also include additional comments for the author, including concerns about dual publication, research ethics, or publication ethics. (Please upload your review as an attachment if it exceeds 20,000 characters)

Reviewer #1: The manuscript is a good study revisiting the PDT therapy for vascular restenosis however the sample size is very low to make a conclusion. There is also not much novelty in the study as this was a study that was performed a while ago and published. There is great potential in exploring the PDT therapy for vascular restenosis however additional pre-clinical data is required with an increased sample size and the use of a different animal model as suggested by the authors in the manuscript. It is unfortunate that there was 40% mortality rate and 70% of the data were unusable.

Reviewer #2: This peer review evaluates the manuscript with a focus on its scientific rigor, relevance to the field, and contribution to ongoing research in vascular photodynamic therapy (PDT). This manuscript presents a thorough reevaluation of a pivotal historical study examining porfimer sodium photodynamic therapy (PDT) for the prevention of vascular restenosis in a rabbit balloon-injury model. The authors provide a data-driven analysis reflecting on efficacy rates, procedural mortality statistics, and methodological outcomes, offering measured insight into the study’s translational potential and the implications for future research in vascular PDT.

*Strengths:-

The study revisits a foundational experiment, reporting efficacy endpoints with statistical detail. For instance, the manuscript describes PDT-treated arteries that demonstrated a consistent and marked suppression of neointimal hyperplasia compared to controls (IMR 0.29 ± 0.07 vs. 1.65 ± 0.55; P < 0.001:- 82% reduction compared to control group), which warrants attention as a measurable therapeutic benefit. This approach has the potential to inform and guide subsequent investigations into vascular PDT therapies, particularly as the field evolves with new technologies and therapeutic strategies.

Procedural mortality:- It is quantified, with the manuscript indicating a high mortality rate (e.g., “out of ten animals, 40% of animals succumbed intraoperatively & 10% post operative). Such transparency is commendable and is essential for accurate risk-benefit evaluation.

The notably high mortality rate associated with the procedure is a significant concern. The authors appropriately emphasize this finding, which raises questions about the safety profile and feasibility of the therapy for wider clinical application.

Methodological Insights:- The paper discusses key methodological factors such as dosing, procedural techniques, and the intrinsic limitations of the rabbit model. This critical perspective strengthens the manuscript and will benefit readers considering future translational studies

**Weaknesses and Suggestions for Improvement:-**

• While the historical context is well established, the manuscript should provide a direct quantitative comparison of efficacy and mortality outcomes with more recent vascular PDT literature and alternative therapies (e.g., “current drug-eluting stent (DES), drug coated ballon DCB restenosis rates & IMR ”). This will position the findings within the broader clinical research landscape.

•The authors report a high procedural mortality, but further breakdown is needed. Please include a table or figure summarising causes of death and the distribution (e.g., “reasoning for intraoperative & postoperative”) and clarify if mortality correlates with specific, negligence of using capnography, PDT parameters (dose, light exposure/distribution, control of laser power, excessive balloon injury, excessive irradiation time, etc.)

•The statistical analysis section could be expanded to include additional details regarding sample size justification and methods used to address potential confounders.

•Consider including a brief discussion on the ethical implications of high-mortality preclinical studies and how modern standards might address these concerns.

Recommendation

Overall, the manuscript offers valuable insights into both the potential and the limitations of porfimer sodium PDT in vascular restenosis therapy. I commend the authors for their balanced and critical approach. Pending minor revisions to expand discussion on safety and translational relevance, I recommend this study for publication

**********

6. PLOS authors have the option to publish the peer review history of their article (what does this mean?). If published, this will include your full peer review and any attached files.

If you choose “no”, your identity will remain anonymous but your review may still be made public.

**Do you want your identity to be public for this peer review?**  For information about this choice, including consent withdrawal, please see our Privacy Policy.

Reviewer #1: No

Reviewer #2: **Yes:** BHAVDIP DOSHI

**********

[NOTE: If reviewer comments were submitted as an attachment file, they will be attached to this email and accessible via the submission site. Please log into your account, locate the manuscript record, and check for the action link "View Attachments". If this link does not appear, there are no attachment files.]

To ensure your figures meet our technical requirements, please review our figure guidelines: https://journals.plos.org/plosone/s/figures

You may also use PLOS’s free figure tool, NAAS, to help you prepare publication quality figures: https://journals.plos.org/plosone/s/figures#loc-tools-for-figure-preparation.

NAAS will assess whether your figures meet our technical requirements by comparing each figure against our figure specifications.

# Author comment: Author response to Decision Letter 1

**DOI:** 10.1371/journal.pone.0350675.r002

17 Mar 2026

Manuscript Number: PONE-D-25-68528

Title: Reappraisal of a historical porfimer sodium photodynamic therapy study for vascular restenosis: Efficacy, high procedural mortality, and methodological insights from a rabbit balloon-injury model

Journal: PLOS ONE

Date: March 2, 2026

Dear Dr. Sojitra and Reviewers,

We sincerely thank the Academic Editor and both Reviewers for their thorough and constructive evaluation of our manuscript. Their comments have substantially strengthened the work. We have addressed every point raised and detailed our responses below. All changes are highlighted in the revised manuscript with tracked changes.

For clarity, each original comment is reproduced in italics, followed by our response and the specific changes made to the manuscript.

RESPONSES TO ACADEMIC EDITOR COMMENTS

Editor Comment 1: Scientific acceptance criteria and model justification

The manuscript presented very old data and over 20 years many scientific acceptance criteria have been changed particularly, justification of animal model and why safety response data should be considered relevant to human, and response of femoral to coronary arteries.

Response: We appreciate this important point. We have expanded Section 4.3 (Contextualizing the Translational Stall) and added a new paragraph in the Discussion to directly address the evolution of scientific standards over the past two decades. We now explicitly discuss (1) how current ARRIVE 2.0 guidelines and 3Rs principles reframe this historical dataset's significance, (2) the acknowledged translational gap between rabbit femoral and human peripheral (not coronary) arteries in terms of vessel diameter, wall composition, and biomechanical environment, and (3) why the documented safety/feasibility failures are arguably more relevant to modern translational science than efficacy endpoints alone. We also clarify throughout that comparisons are made to peripheral (femoral/femoropopliteal) arteries, not coronary arteries, consistent with our stated clinical target. The following sentence has been added to Section 4.3:

Added text (Section 4.3): "It is important to note that the rabbit femoral artery model, while a standard in preclinical vascular biology, carries acknowledged translational limitations relative to human peripheral arteries — including differences in vessel diameter, wall architecture, and susceptibility to inflammatory injury. These differences, in addition to the feasibility failures documented here, must be explicitly acknowledged when interpreting the translational relevance of historical porfimer sodium PDT data. The current report does not claim direct clinical extrapolation, but rather offers methodological lessons for future preclinical studies designed with modern standards."

Editor Comment 2: Unexplained mortality and gross post-mortem evaluation

Furthermore, mortality rate has been higher which remains unexplained. Gross evaluation on non-treated organs and post mortem details would be necessary to appraise those data for readers to repeat experiments without additional animal harm.

Response: We fully agree that this is a critical gap in the original data. We acknowledge candidly that formal post-mortem organ examination (gross pathology of liver, kidneys, lungs, and heart) was not performed on the animals that died intraoperatively or postoperatively — a significant limitation of this historical pilot study. We have added an explicit statement in Section 2.2 (Surgical Procedure) and Section 3.1 (Animal Survival) acknowledging this deficiency and its implications. We have also added a new Table (Table 2: Animal Attrition Summary) in the Results section that provides a structured breakdown of each animal's fate, timing of death, suspected cause, and whether post-mortem data are available.

Added text (Section 3.1): "Formal gross post-mortem organ evaluation was not performed on animals that died intraoperatively or in the early postoperative period — a significant limitation of this historical pilot study. The absence of systematic organ-level data prevents definitive attribution of death to specific causes (e.g., systemic ischemia-reperfusion injury, anesthesia-related cardiac/respiratory failure, or hemorrhage). Future investigators should include structured post-mortem protocols as a mandatory component of any bilateral femoral injury model to enable complete cause-of-death attribution and to inform humane endpoint criteria. This limitation is explicitly noted in Table 2."

Editor Comment 3: Anesthesia protocol explanation

The explanation states that high mortality likely resulted from a confluence of model-specific challenges. Animal death suspected due to anesthesia-related complications is hard to understand, because your lab must have internal protocol anesthesia dose.

Response: We thank the editor for this important challenge. The anesthesia protocol used (intramuscular xylazine 5 mg/kg + ketamine 35 mg/kg, with supplemental half-dose if movement observed) was indeed our institutional protocol at the time (early 2003). However, this combination carries well-documented risks in rabbits, including respiratory depression, cardiovascular suppression, and dose variability due to intramuscular absorption. Critically, our protocol lacked intra-arterial pressure monitoring and capnography — monitoring tools that are now considered standard for major vascular procedures in rabbits. We have substantially revised Section 2.2 to be more explicit about the anesthesia protocol's specific limitations, and Section 4.2 now includes a more nuanced mechanistic discussion of why even a standard-dose protocol may have resulted in high mortality under these specific procedural conditions (bilateral femoral clamping, prolonged surgery, volume shifts).

Revised text (Section 2.2): "General anesthesia was induced using intramuscular xylazine hydrochloride (5 mg/kg) and ketamine hydrochloride (35 mg/kg), which represented the institutional rabbit anesthesia protocol at the time of study (2003). Anesthetic depth was assessed by loss of pedal withdrawal reflex and maintained with a supplemental half-dose upon observed movement. Critically, continuous intra-arterial pressure monitoring and capnography were not employed. These monitoring modalities are now recognized as essential for detecting early cardiovascular compromise during major vascular procedures in rabbits, and their absence represents a significant limitation of the original protocol that likely contributed to the high intraoperative mortality. Body temperature was maintained using a homeothermic blanket. Postoperative analgesia was provided with buprenorphine (0.03 mg/kg, IM) as per institutional guidelines."

Editor Comment 4: Crossover data to add scientific merit

To be more scientific contents, we highly recommend to present any crossover data which can add scientific merit to contents. This data may significantly contribute to final decision.

Response: We interpret "crossover data" as referring to any additional comparative or paired data that could strengthen the biological conclusions. In our within-subject paired design, each animal inherently served as its own crossover control (PDT-treated right/left artery vs. untreated contralateral artery). We have made this paired/crossover structure more explicit in the Methods (Section 2.3) and Results (Section 3.2), including a new supplementary figure (S4 Figure) showing individual animal paired IMR comparisons side-by-side with connecting lines to visualize the within-animal treatment effect. This paired visualization approach — analogous to a crossover display — directly illustrates the consistency of the PDT effect across all three surviving animals and strengthens the biological interpretation. We have also added the individual raw measurement data to the Zenodo repository (updated S2 file).

Editor Comment 5: Histology slides insufficient — full artery views required

Histology slides are insufficient and you should provide full view of artery for all cases (C, D, E and Control).

Response: We fully accept this important request. We have prepared additional high-resolution full cross-sectional H&E images (low-magnification, 4x objective, full vessel lumen view) for all six analyzed artery sections (Animals C, D, and E — both PDT-treated and control sides). These have been added as a new Supplementary Figure (S4: Complete histological panel for all analyzed animals). The original Figure 1 (representative Animal E images at higher magnification with measurement overlay) is retained in the main text. The caption for Figure 1 has been updated to direct readers to the comprehensive S4 panel.

Editor Comment 6: "rats" error in Line 119

Line 119, mentioned rats, you should correct it.

Response: Corrected. Line 119 (Section 2.3) has been changed from "A 425-µm optical fiber with a cylindrical diffuser tip (25 mm) was inserted into the target artery of the rats" to "...the target artery of the rabbits." We apologize for this typographical error.

RESPONSES TO PLOS ONE JOURNAL REQUIREMENTS

Journal Requirement 1: PLOS ONE style requirements

Response: The revised manuscript has been formatted to comply with PLOS ONE style templates. Line numbers have been added throughout, figure file names have been revised to comply with naming conventions, and the title page has been reformatted per the PLOS ONE title/author/affiliation template.

Journal Requirement 2: Animal methods — sacrifice and suffering alleviation

Response: We have expanded the Methods section to include these details. Added to Section 2.2: "Postoperative pain management included buprenorphine (0.03 mg/kg, IM, every 8–12 hours for 48 hours). Animals were monitored twice daily for signs of distress, wound dehiscence, or limb ischemia. Humane endpoints included signs of severe respiratory distress, sustained limb necrosis, or inability to ambulate and access food/water." Added to Section 2.4: "At the 21-day study endpoint, euthanasia was performed by intravenous pentobarbital overdose (100 mg/kg, via marginal ear vein) under deep anesthesia (xylazine/ketamine as described above), consistent with institutional guidelines and the AVMA Guidelines for the Euthanasia of Animals."

Journal Requirement 3: Funder role statement

Response: The funding statement has been updated to include the funder's role. Revised statement: "The Far Eastern Memorial Hospital Research Fund (FEMH 92-D-008) provided financial and administrative support for this study. The funders had no role in study design, data collection and analysis, decision to publish, or preparation of the manuscript." This statement appears in both the manuscript and is provided in this cover letter for the submission form update.

Journal Requirement 4: Full ethics statement in Methods

Response: The full ethics statement has been incorporated into Section 2.1 of the Methods. Revised text: "All procedures were approved by the Institutional Animal Care and Use Committee (IACUC) of Far Eastern Memorial Hospital, New Taipei, Taiwan (Protocol Number: FEMH-92-D-008, approved March 15, 2003). This study was conducted in strict accordance with the recommendations of the Guide for the Care and Use of Laboratory Animals (NIH) and the Taiwan Animal Protection Act. As this study involved no human participants, no human consent was required. All animal surgeries were performed under general anesthesia, and all efforts were made to minimize suffering, as described in Sections 2.2 and 2.4."

Journal Requirement 5: Data availability plan

Response: All raw data are currently deposited and publicly available at Zenodo (https://doi.org/10.5281/zenodo.18054401). The Zenodo repository has been updated with the complete individual animal measurement dataset. The data availability statement in the manuscript has been updated to confirm: "All relevant data are within the manuscript and its Supporting Information files, and the complete raw dataset is publicly available at Zenodo (https://doi.org/10.5281/zenodo.18054401)."

RESPONSES TO REVIEWER #1 COMMENTS

Reviewer #1 Comment 1: Sample size and limited novelty

The manuscript is a good study revisiting the PDT therapy for vascular restenosis however the sample size is very low to make a conclusion. There is also not much novelty in the study as this was a study that was performed a while ago and published. There is great potential in exploring the PDT therapy for vascular restenosis however additional pre-clinical data is required with an increased sample size and the use of a different animal model as suggested by the authors in the manuscript. It is unfortunate that there was 40% mortality rate and 70% of the data were unusable.

Response: We thank Reviewer #1 for this balanced assessment. We agree entirely with the characterization of the sample size limitations and the lack of conceptual novelty — both are acknowledged explicitly in the manuscript as core features of the work rather than flaws to be minimized. The manuscript's primary contribution is not a novel efficacy claim but a transparent feasibility and methodological case study — a genre of scientific reporting increasingly recognized as valuable for the translational research community (per PLOS ONE's own scope statement on "negative and null results"). We have strengthened the framing in the Abstract and Introduction to make this distinction even clearer, so readers immediately understand the study's contribution type.

Specifically, we have revised the Abstract Conclusions to lead with the methodological/feasibility contribution: "This pilot reappraisal does not claim conceptual novelty but provides the scientific community with a complete, transparent historical dataset including documented PDT parameters, a candid feasibility analysis revealing critical translational barriers, and concrete protocol recommendations to guide future investigators — without necessitating additional animal studies."

We also agree with Reviewer #1's recommendation for future studies using larger sample sizes and alternative animal models, and this is now more prominently stated in Section 4.4 (Future Directions), which now explicitly recommends the porcine model as the preferred next step.

RESPONSES TO REVIEWER #2 COMMENTS (Dr. Bhavdip Doshi)

Reviewer #2 Comment 1: Quantitative comparison with recent literature and alternative therapies

While the historical context is well established, the manuscript should provide a direct quantitative comparison of efficacy and mortality outcomes with more recent vascular PDT literature and alternative therapies (e.g., current drug-eluting stent (DES), drug coated balloon DCB restenosis rates & IMR).

Response: We have added a new paragraph in Section 4.3 providing quantitative contextual comparison. Key additions: Contemporary drug-coated balloon (DCB) therapy for femoropopliteal disease (IN.PACT SFA trial, Tepe et al., 2015, reference [24]) shows 12-month primary patency of 82.2% vs. 52.4% for standard PTA. Drug-eluting stents for infrapopliteal disease show restenosis rates of approximately 20–30% at 12 months [Lim et al., 2024, reference [4]]. By contrast, our PDT-treated arteries (in a rabbit model, not directly comparable) showed 82% median IMR reduction at 21 days — a strong biological signal but one that cannot be directly translated to clinical patency rates without further development. This contextualizes the biological promise while maintaining scientific accuracy about the translational gap.

Reviewer #2 Comment 2: Breakdown of causes of death and correlation with procedural parameters

The authors report a high procedural mortality, but further breakdown is needed. Please include a table or figure summarising causes of death and the distribution (e.g., reasoning for intraoperative & postoperative) and clarify if mortality correlates with specific, negligence of using capnography, PDT parameters (dose, light exposure/distribution, control of laser power, excessive balloon injury, excessive irradiation time, etc.)

Response: We have added Table 2 (Animal Attrition and Cause-of-Death Summary) to the

Attachment Submitted filename: 20260303_Response_to_Reviewers_PONE-D-25-685.docx

# Peer review: Decision Letter 1

**DOI:** 10.1371/journal.pone.0350675.r003

**Authors:** Prakash Sojitra

27 Apr 2026

PONE-D-25-68528R1

Reappraisal of a historical porfimer sodium photodynamic therapy study for vascular restenosis: Efficacy, high procedural mortality, and methodological insights from a rabbit balloon-injury model

PLOS One

Dear Dr. Li,

Thank you for submitting your manuscript to PLOS ONE. After careful consideration, we feel that it has merit but does not fully meet PLOS ONE’s publication criteria as it currently stands. Therefore, we invite you to submit a revised version of the manuscript that addresses the points raised during the review process.

Although you have significantly edited manuscript and there few minor comments remains pending.

Please submit your revised manuscript by Jun 11 2026 11:59PM. If you will need more time than this to complete your revisions, please reply to this message or contact the journal office at plosone@plos.org. When you're ready to submit your revision, log on to https://www.editorialmanager.com/pone/ and select the 'Submissions Needing Revision' folder to locate your manuscript file.

Please include the following items when submitting your revised manuscript:

- A letter that responds to each point raised by the academic editor and reviewer(s). You should upload this letter as a separate file labeled 'Response to Reviewers'.
- A marked-up copy of your manuscript that highlights changes made to the original version. You should upload this as a separate file labeled 'Revised Manuscript with Track Changes'.
- An unmarked version of your revised paper without tracked changes. You should upload this as a separate file labeled 'Manuscript'.

If you would like to make changes to your financial disclosure, please include your updated statement in your cover letter. Guidelines for resubmitting your figure files are available below the reviewer comments at the end of this letter.

If applicable, we recommend that you deposit your laboratory protocols in protocols.io to enhance the reproducibility of your results. Protocols.io assigns your protocol its own identifier (DOI) so that it can be cited independently in the future. For instructions see: https://journals.plos.org/plosone/s/submission-guidelines#loc-laboratory-protocols. Additionally, PLOS ONE offers an option for publishing peer-reviewed Lab Protocol articles, which describe protocols hosted on protocols.io. Read more information on sharing protocols at https://plos.org/protocols?utm_medium=editorial-email&utm_source=authorletters&utm_campaign=protocols.

As the corresponding author, your ORCID iD is verified in the submission system and will appear in the published article. PLOS supports the use of ORCID, and we encourage all coauthors to register for an ORCID iD and use it as well. Please encourage your coauthors to verify their ORCID iD within the submission system before final acceptance, as unverified ORCID iDs will not appear in the published article. *Only*  the individual author can complete the verification step; PLOS staff *cannot*  verify ORCID iDs on behalf of authors.

We look forward to receiving your revised manuscript.

Kind regards,

Prakash Sojitra, PhD

Academic Editor

PLOS One

Journal Requirements:

If the reviewer comments include a recommendation to cite specific previously published works, please review and evaluate these publications to determine whether they are relevant and should be cited. There is no requirement to cite these works unless the editor has indicated otherwise.

Please review your reference list to ensure that it is complete and correct. If you have cited papers that have been retracted, please include the rationale for doing so in the manuscript text, or remove these references and replace them with relevant current references. Any changes to the reference list should be mentioned in the rebuttal letter that accompanies your revised manuscript. If you need to cite a retracted article, indicate the article’s retracted status in the References list and also include a citation and full reference for the retraction notice.

Additional Editor Comments:

Response made to reviewers and academic editors are satisfactory

[Note: HTML markup is below. Please do not edit.]

Reviewers' comments:

Reviewer's Responses to Questions

**Comments to the Author**

1. If the authors have adequately addressed your comments raised in a previous round of review and you feel that this manuscript is now acceptable for publication, you may indicate that here to bypass the “Comments to the Author” section, enter your conflict of interest statement in the “Confidential to Editor” section, and submit your "Accept" recommendation.

Reviewer #1: All comments have been addressed

Reviewer #2: All comments have been addressed

**********

2. Is the manuscript technically sound, and do the data support the conclusions?

The manuscript must describe a technically sound piece of scientific research with data that supports the conclusions. Experiments must have been conducted rigorously, with appropriate controls, replication, and sample sizes. The conclusions must be drawn appropriately based on the data presented.

Reviewer #1: Yes

Reviewer #2: Yes

**********

3. Has the statistical analysis been performed appropriately and rigorously?

Reviewer #1: Yes

Reviewer #2: Yes

**********

4. Have the authors made all data underlying the findings in their manuscript fully available?

The PLOS Data policy requires authors to make all data underlying the findings described in their manuscript fully available without restriction, with rare exception (please refer to the Data Availability Statement in the manuscript PDF file). The data should be provided as part of the manuscript or its supporting information, or deposited to a public repository. For example, in addition to summary statistics, the data points behind means, medians and variance measures should be available. If there are restrictions on publicly sharing data—e.g. participant privacy or use of data from a third party—those must be specified.

Reviewer #1: Yes

Reviewer #2: Yes

**********

5. Is the manuscript presented in an intelligible fashion and written in standard English?

PLOS ONE does not copyedit accepted manuscripts, so the language in submitted articles must be clear, correct, and unambiguous. Any typographical or grammatical errors should be corrected at revision, so please note any specific errors here.

Reviewer #1: Yes

Reviewer #2: Yes

**********

6. Review Comments to the Author

Please use the space provided to explain your answers to the questions above. You may also include additional comments for the author, including concerns about dual publication, research ethics, or publication ethics. (Please upload your review as an attachment if it exceeds 20,000 characters)

Reviewer #1: The manuscript titled "Reappraisal of a historical porfimer sodium photodynamic therapy study for vascular

restenosis: Efficacy, high procedural mortality, and methodological insights from a rabbit

balloon-injury model" with the re-submission has addressed all the questions and comments from the previous submission.

Reviewer #2: The author has now put his best effort to address the scientific and methodological concerns raised in the previous version. Statistical analyses have been revised for accuracy and reproducibility, with clearer presentation of outcome measures and mortality rates. The discussion has been expanded to contextualise the study's findings within contemporary vascular PDT literature, highlighting advances since the original feasibility study and identifying gaps for future investigation. The authors have added quantitative comparisons with drug-eluting stents (DES) and drug-coated balloons (DCB), but the discussion could be expanded to include the limitations of PDT compared to these technologies, particularly in terms of clinical adoption and cost-effectiveness. Authorship and institutional affiliations are now appropriately structured, adhering to journal requirements. Typographical and formatting errors in the original version have been corrected.

Based on the corrections and improvements incorporated in the revised manuscript, I recommend that this paper be accepted for publication following a final editorial review for minor formatting consistency and language refinement.

**********

7. PLOS authors have the option to publish the peer review history of their article (what does this mean?). If published, this will include your full peer review and any attached files.

If you choose “no”, your identity will remain anonymous but your review may still be made public.

**Do you want your identity to be public for this peer review?**  For information about this choice, including consent withdrawal, please see our Privacy Policy.

Reviewer #1: No

Reviewer #2: **Yes:** BHAVDIP DOSHI

**********

[NOTE: If reviewer comments were submitted as an attachment file, they will be attached to this email and accessible via the submission site. Please log into your account, locate the manuscript record, and check for the action link "View Attachments". If this link does not appear, there are no attachment files.]

To ensure your figures meet our technical requirements, please review our figure guidelines: https://journals.plos.org/plosone/s/figures

You may also use PLOS’s free figure tool, NAAS, to help you prepare publication quality figures: https://journals.plos.org/plosone/s/figures#loc-tools-for-figure-preparation.

NAAS will assess whether your figures meet our technical requirements by comparing each figure against our figure specifications.

# Author comment: Author response to Decision Letter 2

**DOI:** 10.1371/journal.pone.0350675.r004

1 May 2026

Response to Reviewers — Revision R2

Manuscript: PONE-D-25-68528R1

Title: Reappraisal of a historical porfimer sodium photodynamic therapy study for vascular restenosis: Efficacy, high procedural mortality, and methodological insights from a rabbit balloon-injury model

Journal: PLOS ONE

Dear Dr. Sojitra and Reviewers,

We thank the Academic Editor and both Reviewers for their positive assessment and for the final, minor suggestions to strengthen the manuscript. We have addressed the remaining point raised by Reviewer #2 and have performed a thorough language and formatting polish.

Below is our point‑by‑point response.

Reviewer #2 Comment (remaining suggestion):

The authors have added quantitative comparisons with drug-eluting stents (DES) and drug-coated balloons (DCB), but the discussion could be expanded to include the limitations of PDT compared to these technologies, particularly in terms of clinical adoption and cost‑effectiveness.

Response: We agree that this adds important balance to the translational discussion. We have expanded Section 4.3 with a new paragraph that explicitly compares PDT’s practical limitations (two‑step procedure, skin photosensitivity, specialized light‑delivery equipment, dosimetry challenges) against the clinical‑adoption advantages of DES and DCB (single procedure, no photosensitivity, established reimbursement). This contextualizes why PDT has struggled to compete commercially despite biological efficacy. The new text has been incorporated into Section 4.3 of the revised manuscript.

Additional revisions:

The entire manuscript has been proofread for minor typographical and grammatical errors.

Formatting has been checked for consistency with PLOS ONE guidelines (figure labels, reference style, spacing).

No changes have been made to the data, results, or conclusions.

We believe the manuscript is now ready for formal acceptance. Thank you again for the constructive and efficient review process.

Sincerely,

Ai‑Hsien Adams Li, MD, PhD, MPH

Corresponding Author

Attachment Submitted filename: 20260428 Response to Reviewers-R2.docx

# Editor report: Decision Letter 2

**DOI:** 10.1371/journal.pone.0350675.r005

**Authors:** Prakash Sojitra

17 May 2026

Reappraisal of a historical porfimer sodium photodynamic therapy study for vascular restenosis: Efficacy, high procedural mortality, and methodological insights from a rabbit balloon-injury model

PONE-D-25-68528R2

Dear Dr. Al-Hsien

We’re pleased to inform you that your manuscript has been judged scientifically suitable for publication and will be formally accepted for publication once it meets all outstanding technical requirements.

Within one week, you’ll receive an e-mail detailing the required amendments. When these have been addressed, you’ll receive a formal acceptance letter and your manuscript will be scheduled for publication.

An invoice will be generated when your article is formally accepted. Please note, if your institution has a publishing partnership with PLOS and your article meets the relevant criteria, all or part of your publication costs will be covered. Please make sure your user information is up-to-date by logging into Editorial Manager at Editorial Manager® and clicking the ‘Update My Information' link at the top of the page. For questions related to billing, please contact billing support.

If your institution or institutions have a press office, please notify them about your upcoming paper to help maximize its impact. If they’ll be preparing press materials, please inform our press team as soon as possible -- no later than 48 hours after receiving the formal acceptance. Your manuscript will remain under strict press embargo until 2 pm Eastern Time on the date of publication. For more information, please contact onepress@plos.org.

Kind regards,

Prakash Sojitra, PhD

Academic Editor

PLOS One

Additional Editor Comments (optional):

All comments are now addressed and accepted as is.

Reviewers' comments:

# Editor report: Acceptance letter

**DOI:** 10.1371/journal.pone.0350675.r006

**Authors:** Prakash Sojitra

PONE-D-25-68528R2

PLOS One

Dear Dr. Li,

I'm pleased to inform you that your manuscript has been deemed suitable for publication in PLOS One. Congratulations! Your manuscript is now being handed over to our production team.

At this stage, our production department will prepare your paper for publication. This includes ensuring the following:

* All references, tables, and figures are properly cited

* All relevant supporting information is included in the manuscript submission,

* There are no issues that prevent the paper from being properly typeset

You will receive further instructions from the production team, including instructions on how to review your proof when it is ready. Please keep in mind that we are working through a large volume of accepted articles, so please give us a few days to review your paper and let you know the next and final steps.

Lastly, if your institution or institutions have a press office, please let them know about your upcoming paper now to help maximize its impact. If they'll be preparing press materials, please inform our press team within the next 48 hours. Your manuscript will remain under strict press embargo until 2 pm Eastern Time on the date of publication. For more information, please contact onepress@plos.org.

You will receive an invoice from PLOS for your publication fee after your manuscript has reached the completed accept phase. If you receive an email requesting payment before acceptance or for any other service, this may be a phishing scheme. Learn how to identify phishing emails and protect your accounts at https://explore.plos.org/phishing.

If we can help with anything else, please email us at customercare@plos.org.

Thank you for submitting your work to PLOS ONE and supporting open access.

Kind regards,

PLOS ONE Editorial Office Staff

on behalf of

Dr. Prakash Sojitra

Academic Editor

PLOS One
